# Indolent T Cell Lymphoproliferation of the Gastrointestinal Tract: An Evolving Disease Entity

**DOI:** 10.3390/hematolrep16020018

**Published:** 2024-03-22

**Authors:** Luke Wang, Elaine Koh, Beena Kumar, Michael S. Y. Low

**Affiliations:** 1 Monash Health, Monash Medical Centre, Clayton, VIC 3168, Australia; luke.wang@monashhealth.org (L.W.); elaine.koh@monashhealth.org (E.K.); 2 Department of Anatomical Pathology, Monash Health, Clayton, VIC 3168, Australia; beena.kumar@monashhealth.org; 3 Monash Haematology, Monash Health, Clayton, VIC 3168, Australia; 4 School of Clinical Sciences at Monash Health, Monash University, Clayton, VIC 3168, Australia

**Keywords:** indolent lymphoma, T cell lymphoproliferation, cyclosporine

## Abstract

**Background:** Indolent T cell lymphoproliferation of the gastrointestinal tract is a novel entity recently added to the 2016 WHO classification of lymphoid neoplasms. Classically, these patients demonstrate an immunophenotype consistent with T cell proliferation and can be either CD4-positive or CD8-positive but with a low Ki67 index, highlighting the indolent nature of this disease compared to its more aggressive T cell lymphoma counterparts such as enteropathy-associated T cell lymphoma and monomorphic epitheliotropic intestinal T cell lymphoma. **Methods:** Here, we describe one rare case of such a neoplasm under our care, initially presenting with non-specific signs and symptoms and requiring extensive investigations to diagnose. Available cases in the literature reflect a wide variety of ages and ethnicities affected, and any part of the gastrointestinal sites can be affected, which makes diagnosis difficult and prolonged; however, progression beyond lymph nodes is rare, and prognosis is otherwise favourable, particularly if CD8-positive. The optimal management of these patients remains yet to be defined, given the paucity of available cases currently. The current evidence suggests the utility of steroids, cyclosporine, radiotherapy, and a potential role for JAK inhibitors. **Conclusions:** Our case showed an excellent response to the initial course of steroids, with a subsequent successful transition to cyclosporine, keeping symptoms at bay with ongoing stable disease.

## 1. Introduction

Indolent T cell lymphoproliferation of the gastrointestinal tract (ITLPGI) refers to a new entity described in the fifth edition of the World Health Organisation (WHO) Classification of Haematolymphoid Tumours. Other related T cell lymphoproliferative disorders and lymphomas that involve the gastrointestinal tract include enteropathy-associated T cell lymphoma and monomorphic epitheliotropic intestinal T cell lymphoma; however, the clinical course, histopathology, and prognosis as well as management vastly differ, highlighting the importance of obtaining an accurate diagnosis [1]. 

ITLPGI classically presents with abdominal symptoms, including pain, nausea, vomiting, and diarrhoea, that are often persistent or frequently relapse, with the small bowel and colon being the predominant sites of disease. The depth of invasion is normally superficial, and extraintestinal spread to extranodal organs is rare, though it does occur.

Here, we present a case of a 37-year-old female with persisting abdominal pain associated with nausea, vomiting, and diarrhoea, leading to significant morbidity, managed with corticosteroids and cyclosporine.

## 2. Case Report

A 37-year-old female born in Afghanistan presented to our hospital with a 3-week history of post-prandial abdominal pain associated with nausea, vomiting, diarrhoea, and a weight loss of 10 kg. Her history was significant for endometriosis and previously treated latent tuberculosis (managed with 9 months of isoniazid in Afghanistan). The initial workup revealed a normal full blood examination, liver function tests, inflammatory markers, and a lactate dehydrogenase level within normal limits. A CT scan of the abdomen and pelvis showed diffuse mild thickening of the small bowel, numerous mildly enlarged mesenteric lymph nodes (largest up to 1.4 cm), and splenomegaly (16.1 cm craniocaudal height), which was again seen on a follow-up MRI enterogram for a workup of chronic diarrhoea. Her faecal PCR was unremarkable, and no ova, cysts, or parasites were detected. Her coeliac serology (deamidated gliadin peptide IgG and tissue transglutaminase IgA) was negative, along with her Strongyloidiasis and viral serology (HIV, hepatitis B and C). An autoimmune screen was completed, which demonstrated a negative anti-nuclear antibody, ANCA, and ENA panel.

**Figure 1:**
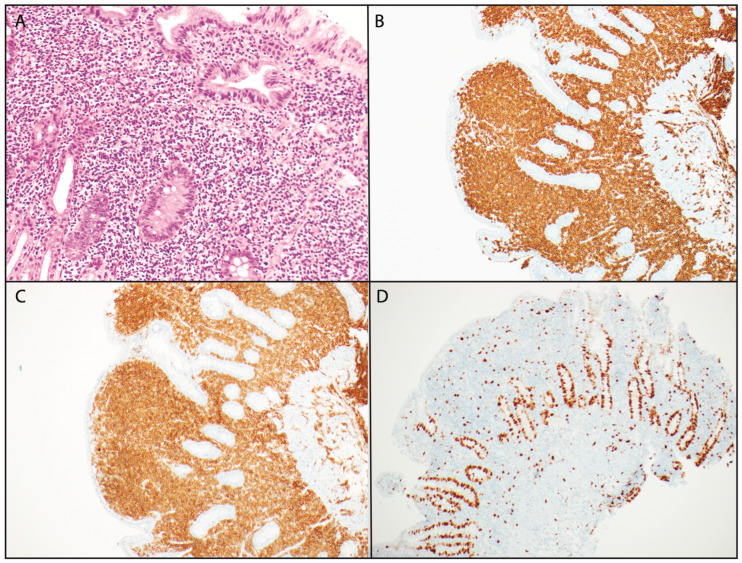
Biopsy of small bowel demonstrating loss of villous architecture and dense lymphoid infiltrate comprising atypical small lymphoid cells and scattered eosinophils (**A**), which in immunohistochemistry stain strongly positive for CD3 (**B**) and CD4 (**C**) with a low Ki-67 index (**D**).

Further workup with an endoscopy revealed macroscopically patchy, friable mucosa in the first portion of the duodenum, and a subsequent colonoscopy was otherwise unremarkable. A biopsy of the small bowel showed dense lymphoid proliferation, comprising mildly atypical lymphoid cells with strong positive immunostaining for CD3, CD4, and CD5 but with a loss of CD7 and CD10 (Figure 1). CD8, CD25, and EBV-ISH staining were all negative. The Ki67 index was low, at 5–10%.

Positron emission tomography demonstrated persisting mesenteric lymphadenopathy but with only mild avidity (SUV ≤ 2), and a mesenteric node biopsy was later completed, showing a similar abnormal lymphoid population to that seen in the duodenum sample. IHC demonstrated diffuse CD3 and CD4 positivity with retained CD5 and a loss of CD7 and CD10. PD1 staining was weak and non-specific. Flow cytometry was like that of the small bowel biopsy, demonstrating a clonal T cell population with CD3, CD4, and CD5 positivity but negative CD7 and CD10.

A bone marrow biopsy was completed, demonstrating an increase in lymphocytes with areas of lymphoid aggregates in the trephine, mainly involving CD3-positive T cells. The flow confirmed a CD3-positive and CD7-negative population, suggestive of marrow involvement in a T cell lymphoproliferative disorder. There were no definitive features of this abnormal population on aspirate.

Our patient was subsequently diagnosed with indolent T cell lymphoproliferation of the gastrointestinal tract, and given her ongoing significant symptoms requiring multiple hospital admissions, she was started on prednisolone 25 mg daily with almost immediate effect in April 2022. Unfortunately, she was unable to be successfully weaned off without a recrudescence of her symptoms, and therefore, she transitioned to cyclosporine 50 mg twice a day in July 2022 with an excellent response.

Interestingly, she had inadvertently self-ceased her cyclosporine for 3 weeks in October 2022, leading to an admission with a relapse of symptoms, including abdominal pain, vomiting, and diarrhoea. These symptoms markedly improved after restarting her cyclosporine, and she has remained on this for several years without significant complications, adverse effects, or a relapse of her disease. 

Annual computed tomography scans have shown stable disease with interval decreases in her splenomegaly and small bowel mesenteric lymphadenopathy since the commencement of therapy in April 2022. She remains under our care with regular outpatient reviews.

## 3. Discussion

Indolent T cell lymphoproliferation of the gastrointestinal tract (ITLPGI) remains rare, with data derived from case series and reports. It is distinct from other T cell lymphomas such as enteropathy-associated T cell lymphoma (EATL) and monomorphic epitheliotropic intestinal T cell lymphoma (MEITL), which typically exhibit aggressive behaviour, culminating in much shorter progression-free and overall survival. EATL is considered a complication of coeliac disease and often preceded by a period of refractoriness to standard therapy, while MEITL has no apparent association with coeliac disease, with a high rate of incidence in the Asian population [2]. Both EATL and MEITL present with symptoms like ITLPGI but macroscopically manifest as ulcerating masses, plaques, or strictures and most commonly affect the small intestine. They typically exhibit high Ki67 indices (>50%), reflecting their rapid proliferative rate and poor prognosis, with the 1-year survivals shown to be 31–39% and 36–39% for EATL and MEITL, respectively [2]. These diseases are difficult to treat, with systemic chemotherapy being the first line of treatment with the possibility of autologous stem cell transplant should they respond, which can improve the 5-year median overall survival. There is also an evolving focus on the role of brentuximab in EATL, given its high rates of CD30 positivity.

ITLPGI itself has been shown to affect a wide variety of ages (15- to 77-year-old) and ethnicities and to have associations with inflammatory bowel disease (IBD), rheumatoid arthritis, and certain viruses, such as HSV and HHV6 [3,4]. However, the underlying aetiology remains unknown. ITLPGI can involve all gastrointestinal sites, most commonly the small intestine and colon, with the disease typically isolated to the mucosa as opposed to aggressive T cell lymphomas [5], and can often appear macroscopically normal, making diagnosis difficult [6]. Additionally, the presenting signs and symptoms are usually non-specific, consisting of chronic abdominal pain, vomiting, diarrhea, and weight loss, which can mimic other primary bowel pathologies such as IBD and coeliac disease.

ITLPGI typically has an indolent clinical course, and disease progression to overt T cell lymphoma is rare but has been seen in several cases [7], with one such case study of a young 37-year-old, who eventually died due to transformation to aggressive T cell lymphoma [8]. Progression is typically seen in CD4 subtypes; however, it is still possible in CD8 variants, as demonstrated in a case study showing transformation into ALK-negative anaplastic large-cell lymphoma in a patient with CD8 ITLPGI [9].

Progression beyond mesenteric lymph nodes is uncommon but does occur, as shown in various case reports demonstrating spread to the spleen, liver, peripheral blood, and bone marrow [5]. The majority of cases are diagnosed immunophenotypically with clonal T cells expressing CD2, CD3, CD5, and CD7 with either CD4 or CD8 positivity. CD56 is typically negative, indicating not a natural killer cell origin. The majority of cases are CD4-positive rather than CD8-positive, which may have a poorer prognostic association [10]. Classically, the Ki67 index is low (<10%), reflecting its typically indolent course [10]. The histopathology often shows a dense, non-destructive infiltration of small tumour cells into the lamina propria, and while extension into muscularis mucosae and submucosa is not unheard of, full thickness involvement has not been seen [10]. The cells themselves demonstrate a mature appearance with minimal atypia. Importantly, it should be noted that, where feasible, diagnosis often requires clonality testing to demonstrate a rearrangement of TCR (αβ or γ) [6].

An evolving field of study is the consideration of an abnormal or inappropriate activation of the STAT signalling pathway as a contributor to the aetiology of ITLPGI, particularly as STAT3 SH2 domain mutations are common in CD8-positive T cell large granular lymphocyte leukaemia, a similar indolent T cell lymphoproliferative disorder [3].

Further evidence to support this has been shown in a subsequent study demonstrating the presence of a STAT3-JAK2 fusion rearrangement using fluorescence in situ hybridization in four of eleven cases with ITLPGI. All four were seen in CD4-positive subtypes and none in CD8-positive or doubly positive subtypes [9]. One case was associated with progression to overt T cell lymphoma.

In vitro, STAT3-JAK2 has been shown to promote the cytokine-independent growth of CD4 T cells through the activation of the STAT pathway, leading to increased transcriptional activity. In this same study, the role of JAK2 inhibitors was also explored, but only in vitro, demonstrating the best efficacy in ruxolitinib (an oral JAK1/2-specific inhibitor with an established role in myeloproliferative neoplasms) when compared to four other various JAK inhibitors with regards to inhibiting the fusion-induced growth of tumour cells [11].

The optimal management of ITLPGI remains unclear, and treatment is often indicated due to debilitating symptoms resulting in significant morbidity [3]. A case series on ten patients with ITLPGI demonstrated the benefit of steroids, which were administered to five of these patients. Four showed a clinical response, two had a partial histological improvement, and three demonstrated long-term stability over 4–8 years [4]. Purine analogues (cladribine, azathioprine) and conventional chemotherapy regimens did not show a therapeutic effect, while interestingly, the use of an anti-CD52 monoclonal antibody induced a clinical response with total radiological remission in two patients [4]. The role of T cell immunosuppressants such as cyclosporine has been hypothesised in the literature given its first-line use in a similar T cell disorder in large granular lymphocytic leukaemia, but there is a lack of case reports demonstrating their successful use in ITLPGI to date [3]. The successful use of radiotherapy has been documented in one such case report with involved field radiotherapy, leading to histological remission upon a repeat biopsy one year later, though this was in only a mildly symptomatic disease localised to the stomach [12]. 

Chemotherapy is generally considered unnecessary and is best demonstrated in a case series where all patients had a non-progressive clinical course, irrespective of whether they were treated with chemotherapy or not [3]. Therefore, the current consensus is to minimise toxicity given the indolent course of the disease and the lack of efficacy or response seen with chemotherapy [4,13].

JAK inhibitors remain a future therapeutic target for ITLPGI given the association between the CD4 variant and STAT3-JAK2 fusion (typically rare in mature T cell neoplasms) [9] and particularly given the worse prognosis associated with CD4-positive ITLPGIs; however, this is yet to be explored in vivo or through clinical trials.

## 4. Conclusions

This case report represents a case of indolent T cell lymphoproliferation of the gastrointestinal tract successfully initially managed with corticosteroids, with sustained clinical remission after transitioning to cyclosporine, with radiological features of regression.

While most cases of indolent T cell lymphoproliferation of the gastrointestinal tract do not progress or advance, the significant morbidity of the condition highlights the importance of diagnosis, particularly given the reported success of various approaches in the literature. Additionally, given its non-specific clinical features, it can be difficult to differentiate from intestinal T cell lymphomas and other primary gastrointestinal disorders such as IBD and coeliac disease, all of which have very contrasting clinical outcomes and approaches to management.

## Data Availability

The data used and analysed during the current study are available from the corresponding author on reasonable request.
